# Development and evaluation of specific polymerase chain reaction assays for detecting *Theileria equi* genotypes

**DOI:** 10.1186/s13071-023-06045-z

**Published:** 2023-11-25

**Authors:** Believe Ahedor, Davaajav Otgonsuren, Atambekova Zhyldyz, Azirwan Guswanto, Noel Muthoni Mumbi Ngigi, Maria Fátima Rodríguez Valinotti, Hemal Kothalawala, Nizanantha Kalaichelvan, Seekkuge Susil Priyantha Silva, Hemali Kothalawala, Tomás Javier Acosta, Thillaiampalam Sivakumar, Naoaki Yokoyama

**Affiliations:** 1 National Research Center for Protozoan Diseases, Obihiro University of Agriculture and Veterinary Medicine, Inada-Cho, Obihiro, Hokkaido 080-8555 Japan; 2 Department of Animal Experimentation, Noguchi Memorial Institute for Medical Research, University of Ghana, Accra, Ghana; 3 Centro de Diagnostico Veterinario, San Lorenzo, Paraguay; 4 Veterinary Research Institute, Peradeniya, Sri Lanka; 5 Department of Farm Animal Production and Health, Faculty of Veterinary Medicine and Animal Science, University of Peradeniya, Peradeniya, Sri Lanka; 6 Department of Animal Production and Health, Peradeniya, Sri Lanka; 7 Universidad Nacional de Canendiyu, Salto del Guaira, Paraguay; 8 Field Center of Animal Science and Agriculture, Obihiro University of Agriculture and Veterinary Medicine, Obihiro, Hokkaido Japan; 9 WOAH Reference Laboratory for Equine Piroplasmosis, National Research Center for Protozoan Diseases, Obihiro University of Agriculture and Veterinary Medicine, Obihiro, Hokkaido Japan

**Keywords:** Equine piroplasmosis, Genotype, Polymerase chain reaction, Specificity, *Theileria equi*

## Abstract

**Background:**

*Theileria equi* causes equine piroplasmosis, an economically significant disease that affects horses and other equids worldwide. Based on 18S ribosomal RNA (18S rRNA sequences), *T. equi* can be classified into five genotypes: A, B, C, D, and E. These genotypes have implications for disease management and control. However, no conventional polymerase chain reaction (PCR) assays are available to differentiate the genotypes of *T. equi*. To overcome this limitation, we developed and evaluated PCR assays specific for the detection of each *T. equi* genotype.

**Methods:**

A pair of forward and reverse primers, specifically targeting the 18S rRNA sequence of each genotype, was designed. The genotype-specific PCR assays were evaluated for their specificity using plasmids containing inserts of the 18S rRNA sequence of each genotype. Subsequently, the assays were tested on 270 *T. equi*-positive equine blood DNA samples (92 from donkeys in Sri Lanka and 178 from horses in Paraguay). 18S rRNA sequences derived from the PCR amplicons were analyzed phylogenetically.

**Results:**

Each genotype-specific PCR assay accurately targeted the intended genotype, and did not produce any amplicons when 18S rRNA from other *T. equi* genotypes or genomic DNA of *Babesia caballi* or uninfected horse blood was used as the template. Previous studies employing PCR sequencing methods identified *T. equi* genotypes C and D in the Sri Lankan samples, and genotypes A and C in the Paraguayan samples. In contrast, our PCR assay demonstrated exceptional sensitivity by detecting four genotypes (A, C, D, and E) in the Sri Lankan samples and all five genotypes in the Paraguayan samples. All the Sri Lankan samples and 93.3% of the Paraguayan samples tested positive for at least one genotype, further emphasizing the sensitivity of our assays. The PCR assays also had the ability to detect co-infections, where multiple genotypes in various combinations were detected in 90.2% and 22.5% of the Sri Lankan and Paraguayan samples, respectively. Furthermore, the sequences obtained from PCR amplicons clustered in the respective phylogenetic clades for each genotype, validating the specificity of our genotype-specific PCR assays.

**Conclusions:**

The genotype-specific PCR assays developed in the present study are reliable tools for the differential detection of *T. equi* genotypes.

**Graphical abstract:**

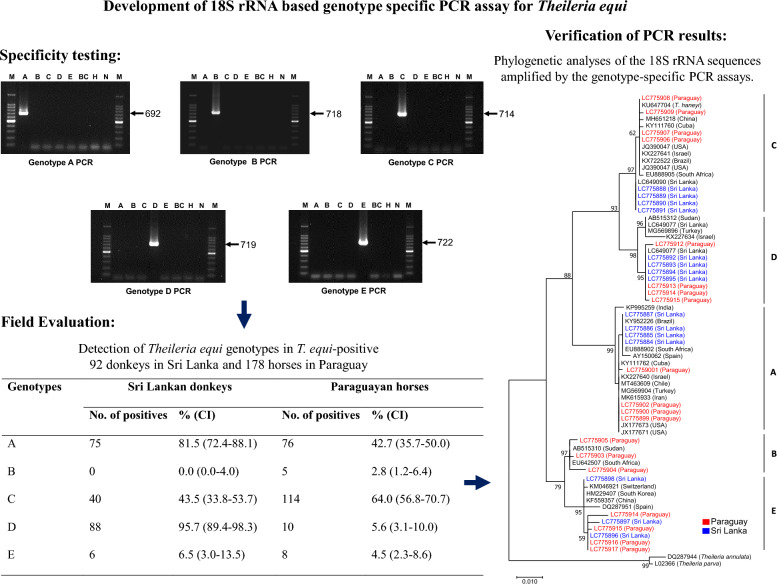

**Supplementary Information:**

The online version contains supplementary material available at 10.1186/s13071-023-06045-z.

## Background

Equine piroplasmosis is a disease caused by *Theileria equi* and *Babesia caballi* in horses and other equids, including mules, donkeys, and zebras, and has a significant economic impact on the equine industry [1–3]. Both parasites have a complex life cycle that involves both equine hosts and tick vectors. In the equine host, *T. equi* and *B. caballi* reproduce asexually within infected erythrocytes, causing massive hemolysis, which leads to severe anemia and other clinical symptoms, such as fever, jaundice, hemoglobinuria, icterus, weight loss, and sometimes death [2]. In general, *T. equi* causes a more severe form of equine piroplasmosis as compared to *B. caballi*. Furthermore, although *B. caballi*-infected animals naturally clear the infection within 4 years, *T. equi*-infected animals become lifelong carriers, from which the infection can be spread to other equines via tick vectors [4–7]. Consequently, *T. equi*-infected carrier animals impede the international equine trade because importing countries, particularly those classified as free from equine piroplasmosis, impose stringent regulations on equine imports [6, 8]. Hence, the control of *T. equi* infection is crucial for an economically sustainable equine industry.

The currently available methods to control *T. equi* infection are heavily influenced by the genetic diversity of the parasite. On the basis of 18S ribosomal RNA gene (18S rRNA) sequences, *T. equi* is classified into five genotypes, named A, B, C, D, and E [9–11]; these can affect the diagnostic results, clinical outcome of infection, and therapeutic efficacy [12–14]. The World Organization for Animal Health recommends competitive enzyme-linked immunosorbent assays (cELISAs) and indirect fluorescent antibody tests [15–18] for the serodiagnosis of *T. equi* infection. cELISA is widely used because of its availability as a kit, predetermined cut-off value that makes the interpretation of test results simple, and suitability for analysis of a large number of samples. The cELISA for the serodiagnosis of *T. equi* infection has been developed based on the equi merozoite antigen 1 (EMA-1) of *T. equi* genotype A [16, 17]. However, a new *Theileria* species, *Theileria haneyi*, which belongs to genotype C of *T. equi*, lacks the *ema-1* gene [10]. Therefore, it is unlikely that the cELISA can detect antibodies in animals infected with the genotype C. The absence of *ema-1* in genotype C also implies that polymerase chain reaction (PCR) assays based on this gene are not suitable for detecting *T. equi* infection. Genotypic diversity of *T. equi* may also influence the outcome of infection, as previous studies have shown that genotype A is more commonly associated with clinical piroplasmosis than the other genotypes [19, 20]. Moreover, the genetic diversity of *T. equi* may be a determinant in drug-induced clearance of the parasite from infected horses, as suggested by the results of a previous study which demonstrated that imidocarb dipropionate eradicates *T. equi* genotype A but not *T. haneyi* [12, 13]. These diagnostic, clinical, and therapeutic implications highlight the importance of detecting the genotype for managing *T. equi* infection and facilitating the safe international transportation of equines.

Currently, PCR sequencing and real-time PCR assays are employed to determine *T. equi* genotypes [21–25]. However, these methods are not without limitations. In the PCR sequencing approach, the 18S rRNA is amplified from *T. equi*-positive samples, sequenced, and phylogenetically analyzed to detect the genotype of the parasite. However, in co-infected animals (i.e., those simultaneously infected with more than one *T. equi* genotype), this method is likely to detect the dominant genotype, potentially leaving the minor genotypes undetected [26, 27]. Real-time PCR assays can provide quick and quantitative results, but they require specialized equipment and expertise [22]. Additionally, the real-time PCR assays available for *T. equi* genotyping have not been validated for wide use in different geographic regions [22, 23]. Because the real-time PCR assays amplify only short fragments of 18S rRNA, validating the findings by sequencing analysis is challenging [22, 23].

On the contrary, conventional PCR assays are more accessible and cost-effective, and can be performed in resource-limited laboratories. Furthermore, the findings can be verified through sequencing analysis [28, 29]. However, conventional genotype-specific PCR assays have not yet been developed for *T. equi*. Therefore, in the present study, we developed 18S rRNA-based PCR assays for the genotype-specific detection of *T. equi*, and evaluated them using previously identified *T. equi*-positive equine DNA samples from Sri Lanka and Paraguay.

## Methods

### Primer design for genotype-specific PCR assays

**Fig. 1:**
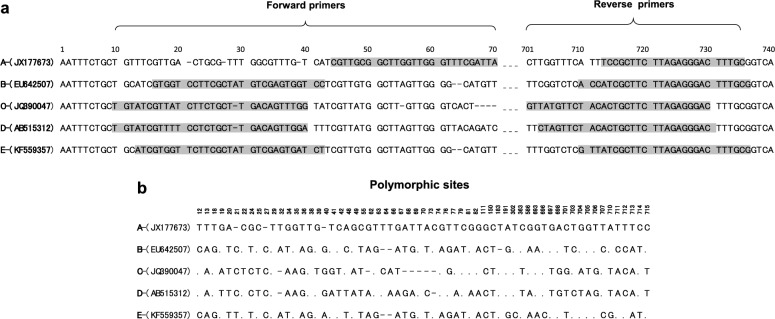
Development of genotype-specific polymerase chain reaction (PCR) assays for *Theileria equi*. **a** Primer design for *T. equi* genotype-specific PCR assays. 18S ribosomal RNA (18S rRNA) gene sequences of *T. equi* were aligned, and a set of forward and reverse primers specific to each genotype was designed. The specific binding regions (highlighted in gray) of the forward and reverse primers on the 18S rRNA sequences representing genotypes A (JX177673), B (EU642507), C (JQ390047), D (AB515312), and E (KF559357) are shown. The dashes represent gaps. **b** Nucleotide polymorphisms among the 18S rRNA sequences targeted by the genotype-specific PCR assays. The 18S rRNA sequences targeted by the PCR assays specific to *T. equi* genotypes A, B, C, D, and E were trimmed and aligned, and the resultant 687-base pair (bp) alignment was analyzed for nucleotide polymorphisms. The dots and dashes represent identical nucleotides and gaps, respectively, to those in the genotype A sequence. Conserved nucleotide polymorphisms in each genotype are shown

Long sequences [~ 1600 base pairs (bp)] of 18S rRNA representing each *T. equi* genotype and those of *B. caballi* and equine hosts were retrieved from GenBank, and aligned using Multalin online software (http://multalin.toulouse.inra.fr/multalin/multalin.html) [30]. On the basis of the alignment, a pair of forward and reverse primers specific to each *T. equi* genotype was designed (Fig. 1a). Moreover, we assessed whether the PCR findings were verifiable through subsequent sequencing analysis of the PCR amplicons. Briefly, the 18S rRNA fragments targeted by the PCR assays specific to genotypes A, B, C, D, and E of *T. equi* were trimmed at both the 5ʹ- and 3ʹ- ends, and aligned. The resulting 687-bp alignment was then analyzed for nucleotide polymorphisms [31].

### Plasmids containing 18S rRNA representing each *T. equi* genotype

Five plasmids, respectively containing an insert of 18S rRNA sequence representing each *T. equi* genotype, were prepared and used for specificity testing of the developed PCR assays. A long 18S rRNA fragment (1591 bp) of genotype A was amplified from DNA extracted from an in vitro culture (*T. equi* USDA strain) [32], using the forward primer Nbab_1F and reverse primer TB-rev [33, 34], as described previously [24]. The 18S rRNA of genotype B was amplified from a DNA template synthesized based on a Sudanese sequence (GenBank accession number AB515312). The resulting PCR amplicons were purified by using a QIAquick Gel Extraction Kit (Qiagen, Hilden, Germany), and then cloned into vector pCR™ 2.1-TOPO (Invitrogen, Carlsbad, CA). The plasmids were purified using a QIAprep Spin Miniprep Kit (Qiagen), and then sequenced. The sequencing analysis confirmed that the 18S rRNA inserts in the plasmids represented the respective genotypes. Plasmids with inserts of 18S rRNA that belong to genotypes C and D were obtained from a donkey survey in Sri Lanka [24], while a plasmid containing 18S rRNA of genotype E was prepared from a horse survey in Mongolia (unpublished data). In the aforementioned studies, the 18S rRNA sequences of genotypes C, D, and E were amplified using the primers Nbab_1F and TB-rev, and then cloned, following the same methodology as in the present investigation.

### Specificity testing

Each PCR assay was evaluated for its ability to differentially detect the target genotype from the remaining genotypes of *T. equi* and DNA of *B. caballi* and noninfected horse blood. Briefly, plasmid DNA of each genotype, *B. caballi* DNA from an in vitro culture (USDA strain), and blood DNA from an uninfected horse were analyzed in each of the genotype-specific PCR assays. Each PCR assay was performed in a 10-μl reaction mixture, which included 1 µl of 0.01 pg/µl plasmid DNA, 5 μl of 2 × PCR buffer (KOD FX Neo; Toyobo, Osaka, Japan), 1 μl of 2 mM deoxynucleoside triphosphates (Toyobo), 0.5 μl of 10 μM forward and reverse primers (Table 1), 0.1 μl of 1 U/μl KOD FX Neo DNA polymerase (Toyobo), and 1.9 μl of double-distilled water. The PCR reaction mixture underwent an initial denaturation at 95 °C for 5 min, followed by 45 cycles, each including a denaturing step at 95 °C for 30 s, an annealing step at the appropriate temperature (see Table 1) for 1 min, and an extension step at 72 °C for 1 min. Following a final elongation step at 72 °C for 7 min, the PCR products were separated on 1.5% agarose gels, stained with MIDORI Green Xtra (Nippon Genetics, Tokyo, Japan), and then illuminated under ultraviolet light. PCR amplicons of the expected band size (Table 1) were considered to be positive for the respective genotype of *T. equi*. The resulting PCR products were cloned, and two clones per genotype were sequenced to confirm that each PCR assay had amplified the targeted fragment of 18S rRNA.

**Table 1:** Primers used in *Theileria equi* genotype-specific polymerase chain reaction assays

Genotypes	Primers (5ʹ–3ʹ)^a^		Annealing temperature (°C)	Amplicon size (base pairs)
	Forward	Reverse		
A	CGTTGCGGCTTGGTTGGGTTTCGATTA	GCAAAGTCCCTCTAAGAAGCGGA	70	692
B	GTGGTCCTTCGCTATGTCGAGTGGTCC	CGCAAAGTCCCTCTAAGAAGCGATGGT	66	718
C	TGTATCGTTATCTTCTGCTTGACAGTTTGG	GTCCCTCTAAGAAGCAGTGTAGAACATAAC	66	714
D	TGTATCGTTTTCCTCTGCTTGACAGTTGGA	GTCCCTCTAAGAAGCAGTGTAGAACTAG	64	719
E	ATCGTGGTTCTTCGCTATGTCGAGTGATCT	CGCAAAGTCCCTCTAAGAAGCGATAAC	72	722

^a^The primers were designed based on the 18S ribosomal RNA of the genotypes of *T. equi*

### Validation of the PCR assays

A total of 270 *T. equi*-positive blood DNA samples from apparently healthy equines, including 92 donkeys from Sri Lanka [24] and 178 horses from Paraguay [35], were used to validate the newly developed genotype-specific PCR assays. Details regarding the gender, age, and breeds of the Paraguayan horses have been provided in previous studies [24, 35]. However, no any additional information is available for the free-roaming Sri Lankan donkeys. The DNA samples from blood samples of Sri Lankan donkeys and Paraguayan horses were extracted using the QIAamp DNA Blood Mini Kit (Qiagen) and DNAzol reagent (Thermo Fisher Scientific, Waltham, MA), respectively, according to the manufacturers’ instructions. Microscopic examination of thin blood smears indicated that 64 of the Sri Lankan donkeys were positive for *T. equi* [24]. Blood smears were not prepared from the surveyed horses in Paraguay [35]. Phylogenetic analyses were performed in previous studies by using 18S rRNA sequences prepared from a selected number of DNA samples, and detected genotypes C and D in the Sri Lankan donkeys [24] and genotypes A and C in the Paraguayan horses [35]. In the present study, all of the 270 samples were screened in each genotype-specific PCR assay. Randomly selected amplicons from Sri Lankan and Paraguayan samples were cloned for each PCR assay, and two clones per amplicon were sequenced.

### Phylogenetic analysis

The Sri Lankan and Paraguayan 18S rRNA sequences derived from the amplicons from each *T. equi* genotype-specific PCR assay, along with those obtained from GenBank, were aligned using MAFFT online software (https://mafft.cbrc.jp/alignment/server/) [36]. The alignment was analyzed using MEGA X software [37] to predict the best nucleotide substitution model based on the lowest Akaike information criterion value. A maximum-likelihood phylogenetic tree was then constructed with MEGA, employing the Tamura-Nei substitution model [38].

## Results

### Development of genotype-specific PCR assays for *T. equi*

**Fig. 2:**
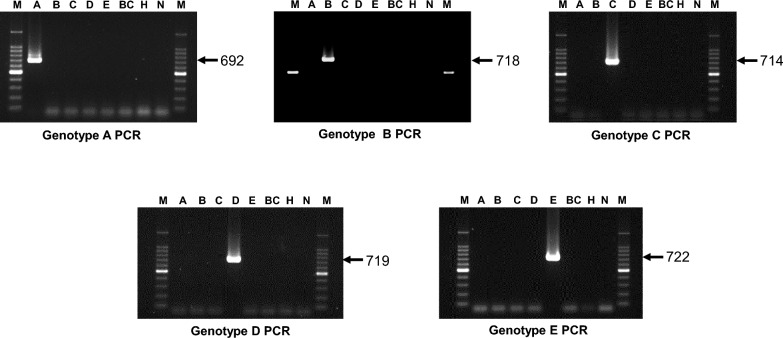
Specificity of the genotype-specific PCR assays for *Theileria equi*. Each genotype-specific PCR assay was evaluated for its ability to differentially detect the target genotype from the remaining genotypes and DNA of *Babesia caballi* and noninfected horse blood. The genotype-specific PCR assays were highly specific in detecting their target genotypes.* A*–*E* Plasmid DNA with inserts of 18S rRNA from *T. equi* genotypes A–E, respectively;* BC* DNA of *B. caballi*;* H* DNA of noninfected horse blood;* N* negative control (no template);* M* 100-bp DNA ladder; for other abbreviations, see Fig. 1

Five 18S rRNA-based PCR assays were developed in the present study for the differential detection of *T. equi* genotypes A, B, C, D, and E. Our in silico analysis showed that the 18S rRNA fragment amplified in each PCR assay is characterized by unique nucleotide polymorphisms, indicating that the results could be confirmed through subsequent sequencing analysis of PCR amplicons (Fig. 1b). The specificities of the genotype-specific PCR assays were evaluated using plasmids with inserts of 18S rRNA representing each *T. equi* genotype. We found that each PCR assay exclusively detected the target genotype, without any amplification observed when 18S rRNA of other genotypes, *B. caballi* DNA, or noninfected horse DNA were tested, thereby validating the specificity of the genotype-specific PCR assays (Fig. 2).

### Field evaluation of genotype-specific PCR assays using *T. equi*-positive equine DNA samples

Our genotype-specific PCR assays were used to screen a total of 270 *T. equi*-positive DNA samples prepared from Sri Lankan donkeys (92 samples) and Paraguayan horses (178 samples). The surveyed donkeys in Sri Lanka were infected with four genotypes of *T. equi*, including A, C, D, and E (Table 2). The most common genotype was D, detected in 88 (95.7%) samples, followed by genotypes A, C, and E, which were detected in 75 (81.5%), 40 (43.5%), and 6 (6.5%) samples, respectively. All 92 *T. equi*-positive donkey samples were positive for at least one genotype (Additional file 1: Table S1). Co-infections were common: 83 (90.2%) donkeys were infected with two, three, or four genotypes (Table 3). Co-infection with genotypes A and D was the most frequently observed combination.

**Table 2:** Detection of *Theileria equi* genotypes in 92 *T. equi*-positive donkeys in Sri Lanka and 178 *T. equi*-positive horses in Paraguay

Genotypes	Sri Lankan donkeys		Paraguayan horses	
	No. of positives	% (95% CI)	No. of positives	% (95% CI)
A	75	81.5 (72.4–88.1)	76	42.7 (35.7–50.0)
B	0	0.0 (0.0–4.0)	5	2.8 (1.2–6.4)
C	40	43.5 (33.8–53.7)	114	64.0 (56.8–70.7)
D	88	95.7 (89.4–98.3)	10	5.6 (3.1–10.0)
E	6	6.5 (3.0–13.5)	8	4.5 (2.3–8.6)

*CI* Confidence interval

**Table 3:** Single and co-infections of *Theileria equi* genotypes in 92 donkeys in Sri Lanka and 166 horses in Paraguay

Genotypes	Number of positives (%)	
	Sri Lankan donkeys	Paraguayan horses
Single genotype		
A	1 (1.1)	46 (27.7)
B	0 (0.0)	2 (1.2)
C	0 (0.0)	75 (45.2)
D	8 (8.7)	2 (1.2)
E	0 (0.0)	1 (0.6)
Total	9 (9.8)	126 (75.9)
Two genotypes		
A + B	0 (0.0)	1 (0.6)
A + C	2 (2.2)	22 (13.3)
A + D	40 (43.5)	0 (0.0)
C + D	8 (8.7)	4 (2.4)
C + E	0 (0.0)	6 (3.6)
D + E	1 (1.1)	0 (0.0)
Total	51 (55.4)	33 (19.9)
Three genotypes		
A + B + C	0 (0.0)	2 (1.2)
A + C + D	27 (29.3)	4 (2.4)
A + C + E	1 (1.1)	1 (0.6)
A + D + E	2 (2.2)	0 (0.0)
Total	30 (32.6)	7 (4.2)
Four genotypes		
A + C + D + E	2 (2.2)	0 (0.0)
Total	2 (2.2)	0 (0.0)
Grand total	92	166

The genotype-specific PCR assays detected all five genotypes in Paraguayan horses. Genotype C was most prevalent, detected in 114 (64.0%) samples, followed by genotypes A, D, E, and B, which were detected in 76 (42.7%), 10 (5.6%), 8 (4.5%), and five (2.8%) samples, respectively. Of 178 T*. equi*-positive horses, 166 (93.3%) were positive for at least one genotype (Additional file 2: Table S2). Of the surveyed horses, 40 (22.5%) had co-infections with two or three *T. equi* genotypes (Table 3); co-infection with genotypes A and C was the most common combination.

**Fig. 3:**
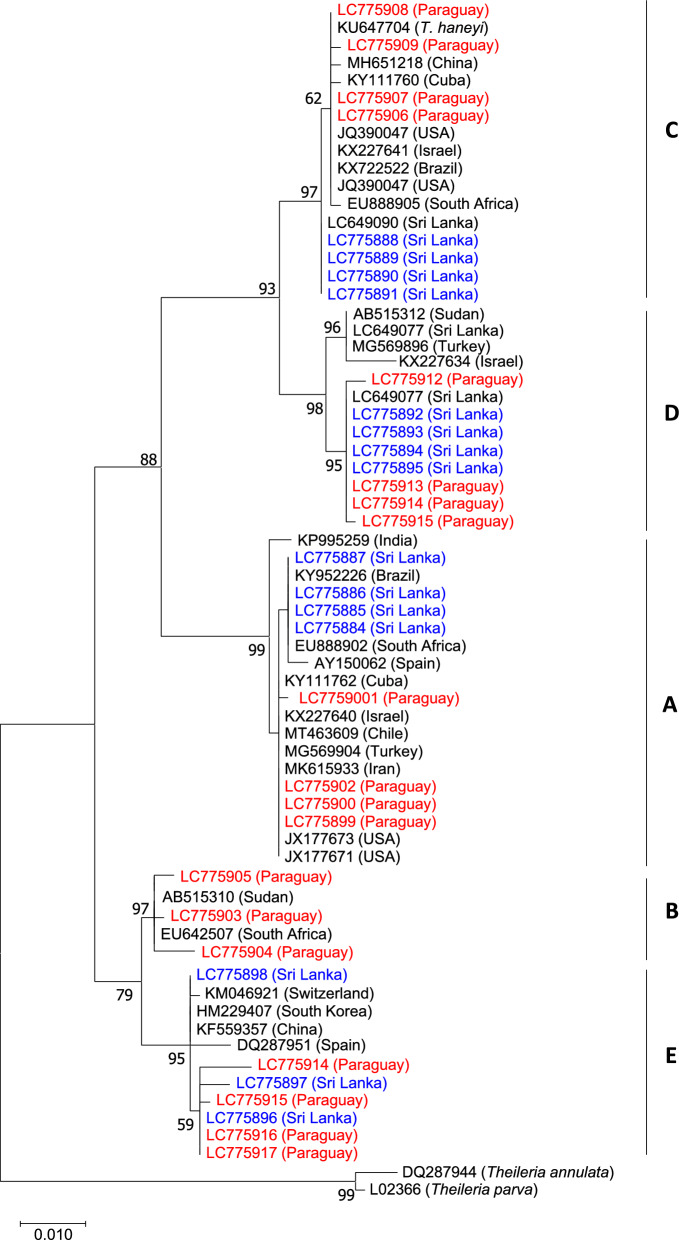
Phylogenetic analysis of *Theileria equi* 18S rRNA. The 18S rRNA sequences amplified in the genotype-specific PCR assays, together with reference sequences representing each genotype, were used to construct a maximum-likelihood phylogeny with 1000 bootstrap replicates. The newly determined sequences from donkeys from Sri Lanka (in red) and horses from Paraguay (in blue) from the amplicons from each genotype-specific PCR assay clustered in the corresponding clades. For abbreviations, see Fig. 1

To validate the PCR results, we sequenced four randomly selected amplicons from each PCR assay, except for genotype E from Sri Lanka and genotype B from Paraguay, for which three amplicons per genotype were sequenced. The resultant 34 18S rRNA sequences, including 15 Sri Lankan sequences representing genotypes A, C, D, and E, and 19 Paraguayan sequences representing all five genotypes, were registered with GenBank (accession nos. LC775884–LC775917) and used in the construction of a phylogenetic tree. All of the newly determined sequences from the respective PCR assays clustered in clades according to their genotype, together with known reference sequences (Fig. 3).

## Discussion

In the present study, we successfully developed a set of five specific PCR assays capable of differentiating the genotypes of *T. equi*. These newly developed PCR assays exhibited high specificity toward their target genotypes, as evaluated using 18S rRNA templates representing each of the genotypes. Subsequently, we validated the assays using *T. equi*-positive DNA samples prepared from donkeys from Sri Lanka and horses from Paraguay, focusing on their sensitivity, specificity, and ability to detect multiple genotypes in co-infected equines. We also investigated whether the findings were verifiable by subjecting the sequences of the PCR amplicons to phylogenetic analysis.

The genotype-specific PCR assays were highly sensitive in detecting the *T*. *equi* genotypes from field samples, as shown by the fact that all of the *T. equi*-positive samples from Sri Lanka and 93.3% of those from Paraguay were positive for at least one genotype. However, a small number of field samples from Paraguay (12/178) tested negative in all five genotype-specific PCR assays. The field DNA samples used in the present study had tested positive in a diagnostic PCR assay using a pair of primers that had been designed based on regions in 18S rRNA that are highly conserved among all *T. equi* genotypes [39]. Therefore, it is possible that the negative samples might have been co-infected with multiple genotypes, each with very low parasitemia, leading to negative results in the genotype-specific PCR assays, while the combined 18S rRNA templates from each genotype could have been sufficient to generate positive results in the diagnostic PCR assay [35].

In the PCR sequencing approach, only a subset of positive samples is subjected to sequencing analysis because of cost and time limitations. As a result, any additional genotypes present in untested samples may remain undetected. By contrast, genotype-specific PCR assays offer a convenient alternative for analyzing a large number of positive samples, and potentially detect all of the genotypes that are present [28, 29]. Previous studies using the PCR sequencing approach identified genotypes C and D in the samples from Sri Lanka and genotypes A and C in those from Paraguay [24, 35]. Notably, our genotype-specific PCR assays not only confirmed the presence of these genotypes, but also detected additional genotypes A and E in the samples from Sri Lanka and B, D, and E in the samples from Paraguay, which have not been previously reported in these countries [24, 35]. The detection of genotype A as the second most common genotype in both Sri Lanka and Paraguay is worrying because this genotype is more commonly associated with clinical piroplasmosis than the others [19, 20]. Similarly, the high rates of detection of genotype C suggest that the currently available cELISA may not be suitable for the serodiagnosis of *T. equi* infection in Sri Lanka and Paraguay [10].

Our PCR assays successfully detected co-infections with multiple genotypes of *T. equi* in both Sri Lankan and Paraguayan DNA samples. The common occurrence of co-infections might have implications for the control of *T. equi*. For example, a previous study found that repeated treatment with imidocarb dipropionate clears the parasites from horses singly infected with genotype A, but not from horses co-infected with genotype A and *T. haneyi* (genotype C) [13]. The ability of the genotype-specific PCR assays to detect co-infections, as demonstrated in the present study, overcomes the limitation of the PCR sequencing approach, which tends to detect only the dominant genotype in co-infected samples. Collectively, our findings demonstrate that these genotype-specific PCR assays are a potential alternative to the PCR sequencing approach for *T. equi* genotyping.

Our genotype-specific PCR assays require validation using a larger number of *T. equi*-positive samples from diverse geographic locations. We found that the 18S rRNA fragments targeted by the genotype-specific PCR assays contain unique nucleotide polymorphisms that can be readily used to verify the PCR results. Furthermore, the 18S rRNA sequences obtained from randomly selected amplicons from each of the genotype-specific PCR assays clustered in their respective phylogenetic clades. These findings not only confirm the specificity of our PCR assays but also suggest that the genotype-specific PCR assays can be validated easily.

The genotype-specific PCR assays developed in the present study have the potential to facilitate further research into the various implications of *T. equi* genotypes, such as for taxonomy, virulence, immunological cross-reactivity, infection persistence, diagnosis, and transmission. A recent study that compared the morphology and whole genome data of genotypes A and C concluded that genotype C represents a novel species, named *T. haneyi* [10], and suggested that several cryptic *Theileria* species have been collectively classified as *T. equi*. Similar comparisons of morphology and genome data among all *T. equi* genotypes are essential to uncover potential novel *Theileria* species [18]. Moreover, the potential for a specific genotype to be a risk factor for clinical piroplasmosis in infected horses remains uncertain. Genotype A appears to be most associated with clinical cases [19, 20], but to confirm this assumption, it would be necessary to comparatively evaluate the virulence of each genotype in experimental infections. Furthermore, not all genotype A infections in horses progress to clinical piroplasmosis [19, 20]. One possibility is that immunity to other genotypes may protect against the disease caused by genotype A, similar to observations with other *Theileria* species [40]. To explore this hypothesis, horses could be experimentally infected with each *T. equi* genotype, and then challenged with genotype A. However, if immunity to *T. equi* is genotype-specific, it would be important to investigate if the persistence of infection arises from co-infections. Monitoring horses infected with single and multiple genotypes for infection persistence could be a simple way to explore co-infections as a reason for infection persistence. Developing novel diagnostic assays capable of detecting all *T. equi* genotypes is vital to overcoming the limitations of current diagnostic methods [41]. However, progress in developing such tests has been slow because of the lack of research materials representing some genotypes. The implications of the genotype for the transmission of *T. equi* also remain uninvestigated. Several tick species belonging to the genera *Amblyomma*, *Dermacentor*, *Hyalomma*, and *Rhipicephalus* transmit *T. equi* [7, 42], but no studies have investigated whether the tick species that transmit *T. equi* are genotype specific. The initial step towards investigating the aforementioned research topics is identifying each genotype in infected hosts. We firmly believe that the genotype-specific PCR assays newly established here, which can be used even in resource-limited laboratories, will prove to be a valuable tool to expedite research focusing on the implications of *T. equi* genotypes.

## Conclusions

The development and evaluation of genotype-specific PCR assays for *T. equi* represent a significant step toward enhancing our understanding of the genotypic diversity and distribution of this important pathogen. These assays offer a sensitive, specific, and efficient approach, suitable even for resource-constrained laboratories, for the differential detection of *T. equi* genotypes. Genotype-specific PCR assays have important implications for disease surveillance, control strategies, and further research in the field of equine piroplasmosis.

### Supplementary Information

**Additional file 1: Table S1.** The distribution of *Theileria equi* genotypes among Sri Lankan donkeys investigated in this study.

**Additional file 2: Table S2.** The distribution of *Theileria equi* genotypes among Paraguayan horses investigated in this study.

## Data Availability

All relevant data generated during the present study are included in this manuscript.

## Abbreviations

cELISA: Competitive enzyme-linked immunosorbent assay
EMA-1: Equi merozoite antigen-1
18S rRNA: 18S ribosomal RNA
